# Essentials of Chemistry and Toxicology

**Published:** 1888-05

**Authors:** 


**Essentials of Chemistry and Toxicology,** for the use of students in medicine. By R. A. WITTHAUS, A. M., M. D., Professor of Chemistry in the University of New York. Second edition. New York: Wm. Wood & Co. 1888.

"There is no short road to knowledge," but books of the character of the above have a place. In reading the larger text-books, it is undoubtedly an aid to the student to have a compend of "Essentials," by which he can review his knowledge, and fix the most important points more firmly in his memory. Both of these books are good, and are well adapted to the end in view.

